# Editorial: Factors influencing user engagement with digital mental health interventions

**DOI:** 10.3389/fdgth.2023.1197301

**Published:** 2023-04-18

**Authors:** Judith Borghouts, Claudette Pretorius, Amid Ayobi, Saeed Abdullah, Elizabeth V. Eikey

**Affiliations:** ^1^ Department of Medicine, University of California Irvine, Irvine, CA, United States; ^2^ Insight Centre for Data Analytics, University College Dublin, Dublin, Ireland; ^3^ UCL Interaction Centre, University College London, London, United Kingdom; ^4^ College of Information Sciences and Technology, Penn State University, University Park, PA, United States; ^5^ Herbert Wertheim School of Public Health and Human Longevity Science, University of California, San Diego, La Jolla, CA, United States; ^6^ The Design Lab, University of California, San Diego, La Jolla, CA, United States

**Keywords:** eHealth, mental health, digital mental health interventions, user experience, user engagement, user adoption

**Editorial on the Research Topic** Factors influencing user engagement with digital mental health interventions

Digital mental health interventions (DMHIs) are a promising solution to increase access to mental health support and overcome barriers to traditional mental health care, including costs (1) and stigma (2, 3). However, despite DMHIs' potential, user engagement with them has been known to vary (4). There are various factors that can impact engagement, such as user-related factors (e.g., type and severity of mental health symptoms), intervention-related factors (e.g., type of content offered by the intervention), and technology- or environment-related factors (5).0

This Research Topic on *Factors Influencing User Engagement with Digital Mental Health Interventions* includes 8 articles to further investigate the various factors influencing user engagement and to what extent current DMHIs take these factors into account.

Using a variety of research methods, and focusing on a range of populations, this Research Topic presents new insights into factors that influence user engagement with DMHIs. For example, older adults may have little confidence or experience using technology (6), women from refugee backgrounds may prioritize inherently social mental health experiences over digital self-care apps (7), and lower-income family members may not have the required resources to access DMHIs, such as high-speed internet and a smartphone (1, 8). Articles in this Research Topic included different populations such as older adults, adolescents and students, Black women, people with schizophrenia, and mental health professionals.

**Raue et al.** explored a message-based psychotherapy tool with 2,470 older adults and matched them with a cohort of younger adults aged 26–35 years to compare any differences in mental health improvements. They found that while cohorts did not differ in anxiety or depressive symptoms at the end of the study, younger adults experienced a quicker reduction in anxiety symptoms. Furthermore, older adults attended more days in treatment, but there were no differences between cohorts in app usage. **McCall et al.** conducted a focus group to inform the design of a mobile app tailored to Black women in supporting anxiety and depression. Recommendations were related to the type of content and features offered by the app, transparency about data and privacy, and creating a sense of community. **Badawi et al.** explored what factors facilitate user engagement with DMHIs among students and adolescents. They found different factors for frequent and infrequent users of DMHIs. Facilitators for frequent users included announcements about upcoming DMHI programs and having access to a variety of DMHI programs. Infrequent users wanted more concrete information about how DMHIs might help them and wanted to monitor their progress when completing DMHI activities.

**Simões de Almeida and Marques** conducted a scoping review of 28 research studies to synthesize factors that may influence how people with schizophrenia engage with DMHIs. The authors identify a number of factors such as providing incentives to engage with the tool and reliability of content. Lastly, **Moilanen et al**. focused on members of the general public and found that people's personal prior experience with DMHIs affected their perceived trust in a chatbot. Overall, participants gave higher security and trust scores to a traditional web interface compared to a chatbot interface. These studies highlight the importance of understanding different user populations when developing and implementing DMHIs and considering their unique needs.

In addition to different types of end-users, mental health providers may have different perspectives on what is needed to facilitate DMHI adoption and engagement. **Feijt et al.** conducted a survey with 1,039 mental health professionals and asked about their use of digital mental health tools. In line with previous work (9), they found the COVID-19 pandemic had led to an increase in the perceived value and use of DMHIs, and the majority of professionals intended to continue to use DMHIs in their practices.

While user studies can provide insight into user needs that may affect user engagement, **Rickard et al.** reviewed 91 DMHIs focusing on five areas: accessibility, integrity, clinical and research evidence base, user engagement, and interoperability. They found that only one DMHI fulfilled all these five evaluation metrics, indicating the need for more robust design and evaluation process in this domain.

People use a mixture of tools to support their health and well-being (10), and it may be cumbersome to add yet another digital tool to this collection. Rather than creating a standalone mental health tool, **Balcombe and de Leo** explored the opportunity to integrate mental health into existing technologies users may already use. In their opinion article, they focused on utilizing a music streaming platform for this purpose. The authors proposed that music platforms can be a useful platform to integrate with mental health, as listening to and creating music can reduce stress, anxiety, and depression. Furthermore, popular streaming platforms like Spotify already use their platform to host playlists and podcasts around mental health, which can reduce stigma around mental health help-seeking. The authors proposed to host the vetted digital mental health platform MindSpot on these streaming platforms in order to assess differences in mental health outcomes as well as user experience and link these outcomes with platform engagement and user behavior.

Overall, the studies in this Research Topic highlight that: a one-size-fits-all approach may not work and designing for specific populations in ways that addresses their unique needs may increase acceptance and engagement; the COVID-19 pandemic has increased use and perceived value of DMHIS among mental health professionals and facilitated their intention for integrating DMHIs into their in-person care; not all publicly available DMHIs are of an equally high-quality standard, highlighting the need to hold app developers accountable to minimum-quality standards and for users and practitioners to assess whether DMHIs meet these standards; there is an opportunity to integrate mental health support into existing digital products.

Researchers and developers can use these findings to inform development and evaluation of DMHIs to create meaningful tools and better understand the contextual factors that impact user engagement with these tools.

## References

[1] Torous J Keris M Myrick J Rauseo-Ricupero N Firth J. Digital mental health and COVID-19: using technology today to accelerate the curve on access and quality tomorrow. JMIR Ment Health. (2020) 7(3):18848. 10.2196/18848 PMC7101061 32213476

[2] Gulliver A Griffiths KM Christensen H. Perceived barriers and facilitators to mental health help-seeking in young people: a systematic review. BMC Psychiatry. (2010) 10(113). 10.1186/1471-244X-10-113 21192795 PMC3022639

[3] Velasco AA Cruz ISS Billings J Jimenez M Rowe S. What are the barriers, facilitators and interventions targeting help-seeking behaviours for common mental health problems in adolescents? A systematic review. BMC Psychiatry. (2020) 20(1). 10.1186/s12888-020-02659-0 PMC7291482 32527236

[4] Baumel A Muench F Edan S Kane JM. Objective user engagement with mental health apps: systematic search and panel-based usage analysis. J Med Internet Res. (2019) 21(9):e14567. 10.2196/14567 31573916 PMC6785720

[5] Borghouts J Eikey EV Mark G De Leon C Schueller SM Schneider M Barriers to and facilitators of user engagement with digital mental health interventions: systematic review. J Med Internet Res. (2021) 23(3):e24387. 10.2196/24387 33759801 PMC8074985

[6] Borghouts J Eikey EV De Leon C Stephen M Schueller SM Schneider M Understanding the role of support in digital mental health programs with older adults: users’ perspective and mixed methods study. JMIR Form Res. (2022) 6(12):e43192. 10.2196/43192 36512387 PMC9795392

[7] Ayobi A Eardley R Soubutts E Gooberman-hill R Craddock I Ann AO. Digital mental health and social connectedness: experiences of women from refugee backgrounds. Proc ACM Hum-Comput Interact. (2022) 6:1–27. 10.1145/3555620

[8] Skorburg JA Yam J. Is there an app for that?: ethical issues in the digital mental health response to COVID-19. AJOB Neurosci. (2021) 13:177–90. 10.1080/21507740.2021.1918284 33989127

[9] Sherrill AM Christopher WW Abdullah S Arriaga RI. Overcoming clinician technophobia: what we learned from our mass exposure to telehealth during the COVID-19 pandemic. J Technol Behav Sci. (2022) 7:547–53. 10.1007/s41347-022-00273-3 36034538 PMC9391067

[10] Burgess ER Zhang R Kiranmai Ernala S Feuston JL De Choudhury M Czerwinski M Technology ecosystems: rethinking resources for mental health. Interactions. (2021) 28:66–71. 10.1145/3434564

